# *TMEM244* Is a Long Non-Coding RNA Necessary for CTCL Cell Growth

**DOI:** 10.3390/ijms24043531

**Published:** 2023-02-09

**Authors:** Karolina Rassek, Katarzyna Iżykowska, Magdalena Żurawek, Monika Pieniawska, Karina Nowicka, Xing Zhao, Grzegorz K. Przybylski

**Affiliations:** 1 Institute of Human Genetics, Polish Academy of Sciences, 60-479 Poznan, Poland; 2 Department of Pathology and Medical Biology, University of Groningen, University Medical Center Groningen, 9700 RB Groningen, The Netherlands

**Keywords:** *TMEM244*, Sézary syndrome, CTCL, lncRNA

## Abstract

Transmembrane protein 244 (TMEM244) was annotated to be a member of the TMEM family, which are is a component of cell membranes and is involved in many cellular processes. To date, the expression of the TMEM244 protein has not been experimentally confirmed, and its function has not been clarified. Recently, the expression of the *TMEM244* gene was acknowledged to be a diagnostic marker for Sézary syndrome, a rare cutaneous T-cell lymphoma (CTCL). In this study, we aimed to determine the role of the *TMEM244* gene in CTCL cells. Two CTCL cell lines were transfected with shRNAs targeting the *TMEM244* transcript. The phenotypic effect of *TMEM244* knockdown was validated using green fluorescent protein (GFP) growth competition assays and AnnexinV/7AAD staining. Western blot analysis was performed to identify the TMEM244 protein. Our results indicate that *TMEM244* is not a protein-coding gene but a long non-coding RNA (lncRNA) that is necessary for the growth of CTCL cells.

## 1. Introduction

The transmembrane protein (TMEM) family comprises proteins that are embedded in the cell membrane and span both intracellular and extracellular environments. TMEMs are components of various cell membranes, such as mitochondrial membranes, Golgi membranes, lysosomes, and the endoplasmic reticulum [1]. They are involved in many cellular processes, such as the transport of ions and molecules across impermeable membranes, membrane trafficking, and signaling transduction pathways [2]. Studies showed that TMEMs’ expression can be down- or upregulated in several cancers [3,4] and is associated with tumor progression, disease stage, and patient survival [5,6]. Because membrane proteins are involved in essential cellular pathways, they are often targets of pharmaceutical agents [7]. Yet, for most TMEMs, the mechanism of their involvement in carcinogenesis is still unknown.

The *TMEM244* gene is located in chromosome 6q22.33 and comprises five exons. Under physiological conditions, *TMEM244* is expressed at a low level in the brain and the pituitary glands. To date, the existence of the TMEM244 protein has not been experimentally demonstrated, but the gene is supposed to encode a protein of 128 amino acids and a molecular mass of 14,657 Da. Izykowska et al., were the first to pay attention to the *TMEM244* gene, as it was identified among four other genes (*EHD1*, *MTMR2*, *RNF123*, and *TOX*) to be involved in the rearrangements affecting gene expression in Sézary syndrome (SS) patients compared to controls [8]. Further studies showed that *TMEM244* is expressed in T-cell lymphomas as a result of specific hypomethylation of its promoter, and this expression is associated with poor overall survival in T-cell lymphoma patients [9,10]. A significantly higher expression of *TMEM244* was identified in Sézary syndrome patients, not only compared to healthy individuals but also to SS clinical mimickers, such as mycosis fungoides and erythrodermic manifestations of non-malignant diseases, therefore indicating its diagnostic potential. Moreover, higher expressions of *TMEM244* in CD4+ and CD8+ subsets of memory cells (CD45RO+) were identified, which is in line with the immunophenotype of Sézary cells [11]. The purpose of this study was to establish the function of the *TMEM244* gene, which has not been investigated yet.

## 2. Results

### 2.1. TMEM244 Has a Low Protein-Coding Potential and Cannot Be Detected at the Protein Level

For the detection of the TMEM244 protein, two CTCL cell lines were examined: SeAx and HH, as well as other non-CTCL cell lines, HDLM2, D341med, and COLO684, with predicted high levels of the TMEM244 transcript.

**Figure 1:**
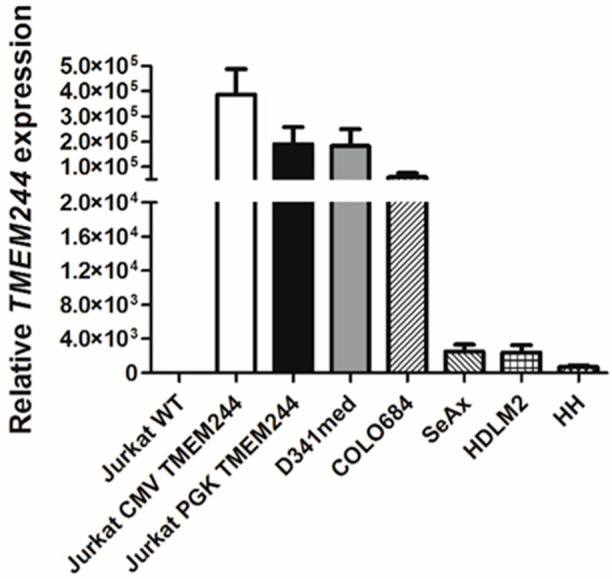
Relative *TMEM244* expression analyzed using RT-qPCR in cell lines with endogenous expression and in cell lines stably transduced with *TMEM244* ORF vectors. The expression was normalized to beta-2-microglobulin. Data are represented as mean ± SD (*n* = 3).

A high level of *TMEM244* expression in the Jurkat cell line was induced using two lentiviral systems with different promoters (human cytomegalovirus; CMV or 3-phosphoglycerate kinase; PGK). FLAG tags were introduced either at the N- or C-terminus of the *TMEM244* gene. *TMEM244* expression on the mRNA level, as determined using RT-qPCR, was the highest in Jurkat-CMV (mean = 390 × 10^3^ ± 102 × 10^3^) and Jurkat-PGK (mean = 190 × 10^3^ ± 69 × 10^3^). In cell lines with endogenous *TMEM244* expression, the highest level was detected in D341med (mean = 180 × 10^3^ ± 67 × 10^3^), followed by COLO684 (mean = 59 × 10^3^ ± 16 × 10^3^), SeAx (mean = 2.5 × 10^3^ ± 0.82 × 10^3^), HDLM2 (mean = 2.4 × 10^3^ ± 0.88 × 10^3^), and HH (mean = 0.7 × 10^3^ ± 0.2 × 10^3^) (Figure 1). Upon confirmation of the expression of *TMEM244* in selected cell lines, Western blot analysis using a custom-made anti-TMEM244 antibody and an anti-FLAG antibody, with a wild-type cell line used as a control, was performed.

Moreover, commercially available brain lysate was analyzed for TMEM244 protein expression, as according to the Human Protein Atlas database *TMEM244* is expressed on the mRNA level in the brain (https://www.proteinatlas.org/ENSG00000203756-TMEM244/tissue; accessed on 30 January 2020). All Western blot analyses showed signals from positive controls, but they failed to detect the TMEM244 protein (Figure S1). The TMEM244 protein was neither detected with the anti-FLAG antibody in cell lines with induced *TMEM244* overexpression (Figure S1A) nor with the specific anti-TMEM244 antibody in the same cell lines, and it was not detected in cell lines or brain tissue with high endogenous *TMEM244* expression (Figure S1B,C). Furthermore, *in silico* analysis showed a very low coding probability of *TMEM244* [0.097], with a cutoff <0.364 indicating a noncoding sequence [12]. Taken together, these results indicate that *TMEM244* is not expressed at the protein level.

### 2.2. TMEM244 Transcript Is Primarily Localized in the Cytoplasm

**Figure 2:**
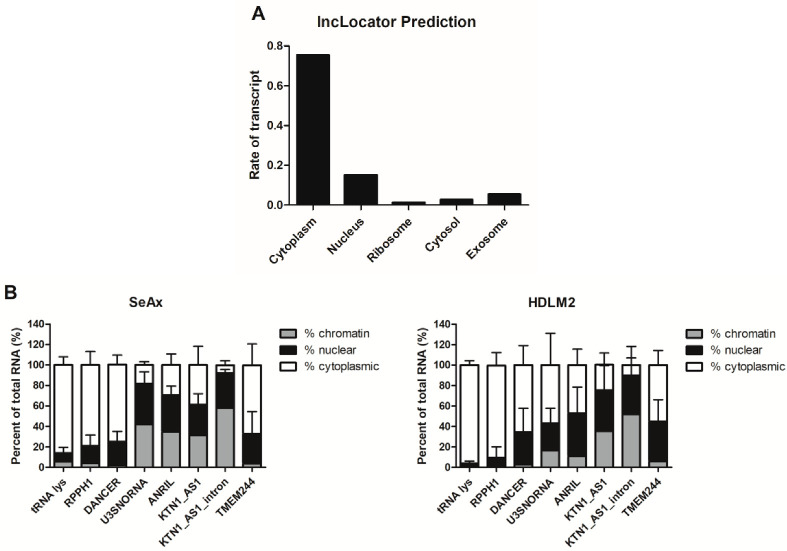

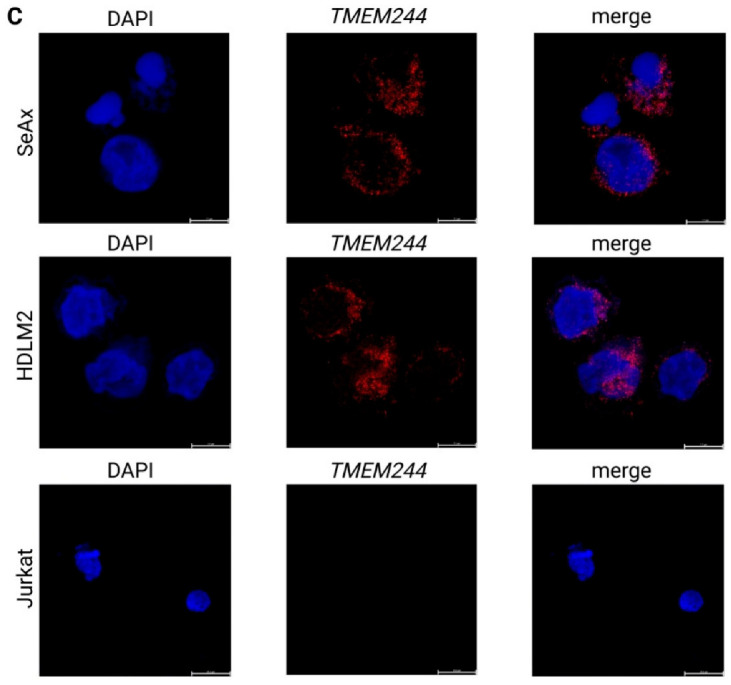
Localization of *TMEM244* transcript. (**A**) lncLocator prediction; (**B**) subcellular fractionation and RT-qPCR analysis of *TMEM244* expression in SeAx and HDLM2 cells; tRNA lys, RPPH1, and DANCER were used as cytoplasmic controls; U3SNORNA and ANRIL-nuclear controls; KTN1_AS1_ and KTN1_AS1_intron-chromatin controls. The mean values ± SD of 3 independent experiments are shown (**C**) FISH analysis of *TMEM244* in SeAx, HDLM2 and Jurkat cells (negative control); *TMEM244* FISH signal in red, DAPI counterstain in blue.

Using the long non-coding RNA subcellular localization predictor (lncLocator; http://www.csbio.sjtu.edu.cn/bioinf/lncLocator/; accessed on 2 September 2021), the *TMEM244* transcript was predicted to be present, mainly in the cytoplasm and partially in the nucleus (Figure 2A). To confirm this prediction, subcellular fractionation was performed in SeAx and HDLM2 cell lines and FISH RNA analysis in SeAx and HDLM2 cells. In both cell lines, the level of the *TMEM244* transcript in the cytoplasm (SeAx = 67%; HDLM2 = 55%) was higher than that observed in the nucleus (SeAx = 29%; HDLM2 = 39%) (Figure 2B). FISH results further confirmed that in SeAx and HDLM2 cells, the prominent distribution of *TMEM244* was in the cytoplasm (Figure 2C).

### 2.3. Inhibition of TMEM244 Results in Decreased Cell Growth in CTCL Cell Lines

**Figure 3:**
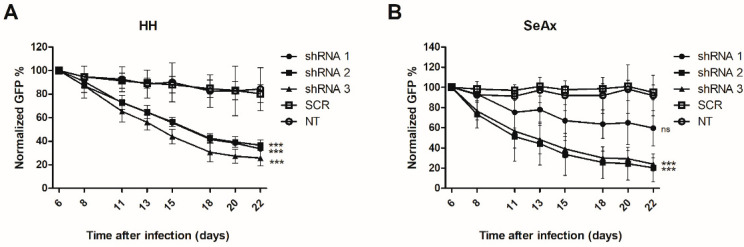
Green fluorescent protein (GFP) growth competition assay with shRNAs targeting *TMEM244* in (**A**) SeAx and (**B**) HH. The effect of *TMEM244* knockdown on cell growth was assessed by following the percentage of GFP+ cells for 22 days post-transduction, with the GFP percentage normalized to day six (*n* = 3); *** *p* < 0.001, ns—non-significant, based on mixed model analysis; NT—non-targeting, SCR—scrambled.

To establish the function of *TMEM244* in cancer cells, the effect of *TMEM244* knockdown was analyzed in SeAx and HH cell lines with endogenous *TMEM244* expression. The effectiveness of the shRNAs targeting the *TMEM244* transcript was confirmed for both cell lines (Figure S2). *TMEM244* silencing resulted in a strong negative effect on cell growth. On day 22 after transduction, the ratio of GFP-positive cells decreased by more than 50% in the cell lines treated with *TMEM244*-specific shRNAs compared to non-targeting and scrambled controls (Figure 3).

The knockdown of *TMEM244* decreased in the GFP+ cell population for all constructs in both CTCL cell lines. In HH, all shRNAs showed a strong effect, with a reduction of 57%, 55%, and 68% for shRNA 1, shRNA 2, and shRNA 3, respectively, compared to SCR and NT. The effects on the growth of SeAx cells were stronger for shRNA 2 and shRNA 3, with a reduction of 78% and 74% compared to SCR, and only a mild effect was observed for shRNA 1 (36%).

**Figure 4:**
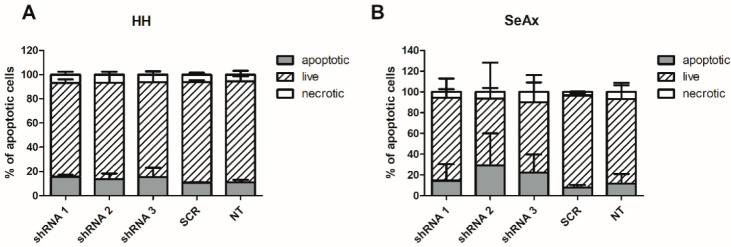
Cell viability upon *TMEM244* knockdown in (**A**) HH; (**B**) SeAx cells. The percentages of apoptotic, live, and necrotic cells were determined using flow cytometry with Annexin V/7AAD staining. The mean values ± SD of 3 independent experiments are shown. NT—non-targeting, SCR—scrambled.

To further investigate the mechanism of growth inhibition upon *TMEM244* knockdown on cancer cells, Annexin V/7AAD staining was conducted (Figure 4).

Only a slight, statistically non-significant difference in the number of apoptotic cells in the HH cell line was detected. In SeAx, although also non-significant, the effect was stronger, with an increase of 7% and 3% using shRNA 1; 21%, and 18% using shRNA 2; and 14% and 11% using shRNA 3, compared to SCR and NT, respectively.

### 2.4. Identification of Novel Alternative Transcripts of TMEM244

**Figure 5:**
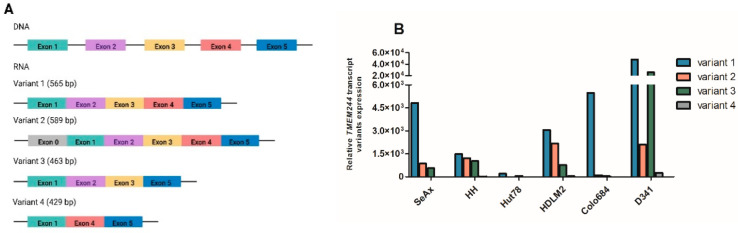
*TMEM244* transcript variants identified with RACE method. (**A**) Transcript variant length scheme; (**B**) relative expression of *TMEM244* transcript variants analyzed by RT-qPCR.

This study led to the identification of two novel alternative *TMEM244* transcripts. Besides the two known transcripts—variant one with 5 exons (RefSeq NM_001010876; ENST00000368143.6) and variant two with an extra 5′ exon (ENST00000438392.2), available in the Genome Browser—two additional variants were identified: variant three without exon 4 and variant four without exons 2 and 3 (Figure 5A and Figure S4). The expression profile of each splice variant in cell lines with *TMEM244* expression (SeAx, HH, Hut78, HDML2, and D341med) was performed using RT-qPCR with variant-specific primers. In most cell lines, the expression level was as follows; variant1 > variant2 > variant3 > variant4, except for D341med, where the expression of variant three was higher than that of variant two (Figure 5B).

To assess the coding potential of each transcript variant, the *in silico* analysis was performed using CPAT. The analysis confirmed that neither of the detected variants had the potential to encode a protein. The coding potential was 0.0976, 0.0003, 0.0359, and 0.0043 for variants one, two, three, and four, respectively.

## 3. Discussion

*TMEM*s are a very heterogeneous group of more than 300 genes, which have been included based on the *in silico* analysis of their DNA sequence. To be annotated as a TMEM gene, the predicted protein structure must contain at least one putative transmembrane segment that spans completely or partially through biological membranes [13]. Some TMEMs have been experimentally shown to encode a protein and, upon functional characterization, have been renamed and reclassified [14]. Still, for many of them, including *TMEM244*, neither the protein nor the function have been experimentally demonstrated.

Our study demonstrated that despite its annotation, based only on the in silico analysis of the predicted protein structure, the *TMEM244* gene does not seem to encode a protein but, rather, belongs to the long-non-coding RNA (lncRNAs) family. LncRNAs are defined as ≥200 nucleotides long RNAs that are spliced and polyadenylated like mRNAs; however, they lack protein-coding activity. *In silico* analysis revealed minor coding potential of the 545 nucleotides long *TMEM244* transcript. *TMEM244* has an open reading frame (ORF) but, as shown for other lncRNAs, such as LINC00116 or LINC00948, possessing an ORF does not determine protein production [15,16]. *TMEM244*, like lncRNAs, is poorly conserved and its expression level is lower compared to protein-coding genes [17]. In addition, the *TMEM244* mRNA level is highest in the brain and pituitary glands, which is typical for lncRNAs.

While mRNAs are very specifically located on the ribosomes in the cytoplasm, lncRNAs may occupy diverse sites, including chromatin, subnuclear domains, nucleoplasm, and cytoplasm [18]. Furthermore, in tumors, the cellular localization of lncRNAs is related to their functions. LncRNAs located in the nuclear compartment usually control transcription and post-transcriptional processing. Since *TMEM244* is mainly located in the cytoplasm, this suggests its involvement in the regulation of translation, mRNA turnover, protein stability, sponging of cytosolic factors, and the modulation of signaling pathways [15,19].

Recently, emerging evidence showed that lncRNAs could promote cell proliferation and, therefore, be engaged in carcinogenesis. For instance, the lncRNA HOXD cluster antisense RNA 1 (*HOXD-AS1*) was upregulated and promoted cell proliferation in cervical cancer, while lncRNA *EPIC1* promoted proliferation and inhibited apoptosis of gallbladder cancer cells [16,20]. LncRNAs can also affect apoptosis by acting as a competitive endogenous RNA (ceRNA) for miRNA and binding to the sequence at the 5′ end of the miRNA, therefore reducing target mRNA expression and ultimately affecting cell apoptosis. Moreover, lncRNAs can act directly or indirectly on death receptors [16]. Previous studies have demonstrated that in some cases, a non-protein-coding locus can give rise to functionally distinct transcript isoforms [21,22,23,24]. Recently, it was shown that the switch in the lncRNA HOTAIR start site after the induction of differentiation promotes the inclusion of HOTAIR exon 3, containing a protein-binding domain, which likely changes its function [25]. We showed that *TMEM244* is necessary for the growth of cells where its expression is at a relatively high level, such as in CTCL cell lines. However, the mechanism behind this observation is still unknown.

It is, however, worth mentioning that the experiments were focused on the sense strand of *TMEM244* based on the GenBank (NCBI) transcription annotation (Gene ID: 253582), and it is not known if the anti-sense strand of *TMEM244* is expressed or whether it plays any role in cell proliferation.

## 4. Materials and Methods

### 4.1. Cell Lines

Seven established cell lines were included in the study. Four were lymphoid cell lines: HH—established from an aggressive cutaneous T-cell leukemia/lymphoma patient (ATCC CRL-2105), SeAx—the Sézary syndrome cell line, kindly provided by Markus Möbs [26], Jurkat—a T-cell acute lymphoblastic leukemia (T-ALL) cell line (88042803; Merck KGaA, Darmstadt, Germany), and HDLM2—a T-cell Hodgkin lymphoma cell line (DSMZ ACC17). In addition, three non-lymphoid cell lines were used: D341 med—a medulloblastoma cell line (ATCC HTB-187), COLO 684—human uterus adenocarcinoma (ECACC 87061203), and HEK293T (DSMZ ACC 635). CTCL and COLO 684 cell lines were cultured in a HEPES-buffered RPMI1640 medium with L-glutamine (Thermo Fisher Scientific™, Waltham, MA, USA), 10–20% fetal bovine serum (FBS) (Merck KgaA, Darmstadt, Germany), and 1% penicillin/streptomycin (Thermo Fisher Scientific™, Waltham, MA, USA), according to the manufacturer’s instructions. The medium for SeAx was supplemented with Il-2 (200 U/mL) (Merck KgaA, Darmstadt, Germany) and the medium for Jurkat with 1% sodium pyruvate (1 mmol/L) and 0.25% glucose (0.5 g/L) (Thermo Fisher Scientific™, Waltham, MA, USA). The D341 med cell line was cultured in Eagle’s Minimum Essential Medium (ATCC 30-2003™), supplemented with 20% FBS (Merck KgaA, Darmstadt, Germany), according to the manufacturer’s protocol. HEK293T cells were cultured in Dulbecco’s Modified Eagle Medium (DMEM) (Lonza, Basel, Switzerland) with 10% FBS (Merck KgaA, Darmstadt, Germany) and 1% penicillin/streptomycin (Thermo Fisher Scientific™, Waltham, MA, USA).

### 4.2. Fluorescence In Situ Hybridization (FISH) Assay

Fluorescence-labeled probes for *TMEM244* and *GAPDH* RNA were designed and synthesized, and FISH experiments were performed according to the manufacturer’s protocol, using the Stellaris™ FISH technology kit (Biosearch Technologies, Hoddesdon, UK). Twenty Quasar^®^ 570-labeled probes for the *TMEM244* transcript were designed using Stellaris^®^ Probe Designer version 4.2 (LGC Biosearch Technologies, Berlin, Germany). The nuclei were stained with DAPI, and Human GAPDH with the Quasar^®^ 570 Dye Stellaris^®^ FISH Probe was used as a cytoplasmic marker (Figure S3). Images were acquired using a Leica DMI8 laser-scanning confocal microscope (Leica Microsystems, Wetzlar, Germany). Cells were imaged with an HC PL APO CS2 100×/1.40 oil objective lens and processed using Leica Application Suite X software (Leica Microsystems, Wetzlar, Germany). All samples were imaged under the same optical conditions.

### 4.3. Generation of Cells with Knockout or Stable Expression of TMEM244

In brief, lentiviral vectors were co-transfected with 3rd-generation packaging plasmids—pMSCV-VSV-G, pRSV.REV, and pMDL-gPRRE—into HEK293T cells using Lipofectamine 2000 (Thermo Fisher Scientific™, Waltham, MA, USA). At 24 h post-transfection, the medium was replaced. At 48 and 72 h post-transfection, viral supernatant was collected, sterile filtered through a 0.45 μm syringe filter, and stored at −80 °C. For *TMEM244* knockdown miRZIP, KLHL6 plasmid was used. Three short hairpins RNAs were designed using the Broad Institute program (https://portals.broadinstitute.org/gpp/public/seq/search; accessed on 13 December 2021) to knock down TMEM244 (shRNA 1 on exon 3, sense: GATCCATCCCTCATGGCTCAACATAATTCAAGAGATTATGTTGAGCCATGAGGGATTTTTTG, antisense: AATTCAAAAAATCCCTCATGGCTCAACATAATCTCTTGAATTATGTTGAGCCATGAGGGATG; shRNA 2 on exon 4, sense: GATCCGAAGAATGGGTTTGGGATTATTTCAAGAGAATAATCCCAAACCCATTCTTCTTTTTG, antisense: AATTCAAAAAGAAGAATGGGTTTGGGATTATTCTCTTGAAATAATCCCAAACCCATTCTTCG; shRNA 3 on exon 5, sense: GATCCGTGGGCTGCTTTAGGTATATCTTCAAGAGAGATATACCTAAAGCAGCCCACTTTTTG, antisense: AATTCAAAAAGTGGGCTGCTTTAGGTATATCTCTCTTGAAGATATACCTAAAGCAGCCCACG) (Figure S5). Control NT2 and SCR vectors were a kind gift from Prof. Anke van den Berg and Dr. Joost Kluiver [27]. Jurkat cells were transduced with lentiviral vectors: pLV-CMV-Tmem244 (flag, 6xHis), pLV-hPGK-Tmem244 (flag, 6xHis), and pLV_flag_CMV_Tmem244. Virus supernatant was added to cells together with polybrene (4 μg/mL). Seax, HH, and D341med cells were transduced with lentiviral vector miRZIP KLHL6. To validate the *TMEM244* overexpression level, cells were infected, aiming at an infection percentage of >70%. To establish a pure population of cells, selection with puromycin was performed for 5–7 days (2 μg/mL). The efficiency of the transduction was measured by flow cytometry using the green fluorescent protein (GPF) signal. Cells were harvested for RNA and protein.

### 4.4. Western Blot

Whole cell lysates were prepared from 5–10 × 10^6^ cells. Cells were washed with phosphate-buffered saline (PBS) and lysed in RIPA buffer (Merck KgaA, Darmstadt, Germany) containing 1X protease inhibitor cocktail (Bioshop Canada Inc., Burlington, ON, Canada) for 30 min on ice. Samples were centrifuged at 14,000× *g* for 30 min to remove DNA or debris. As an additional control, MG132 proteosome inhibitor (Merck KgaA, Darmstadt, Germany) was added to the cell cultures to prevent possible TMEM244 degradation. After 6 h incubation with 1 μM MG132 or 4 h incubation with 20 μM of MG132, cells were washed with phosphate-buffered saline (PBS) and lysed in RIPA buffer (Merck KgaA, Darmstadt, Germany) containing 1X protease inhibitor cocktail (Bioshop Canada Inc., Burlington, ON, Canada) for 30 min on ice. Samples were then sonicated (3 cycles, ON 20 s, OFF 30 s). Total protein concentrations of the cell extracts were measured using the Pierce BCA Protein Quantitation kit (Thermo Fisher Scientific™, Waltham, MA, USA), and the samples were stored at −80 °C until assayed. Prior to loading on gel, samples were heated at 95 °C for 5 min in a heating block. A synthetic peptide-fragment of the putative TMEM244 protein used for mice immunization was used as a positive control. Not centrifuged and nonheated proteins were used as controls for the sample preparation procedure.

Human brain whole tissue lysate was commercially available (Novus Biologicals LLC a Bio-Techne Brand, Centennial, CO, USA). Western blotting was performed as previously described [9]. Primary antibodies (anti-FLAG (F1804, 1:1000, Merck KGaA, Darmstadt, Germany), anti-TMEM244 (custom-made; 1:1000, Proteogenix, Schiltigheim, France)) were used, as well as HRP-labeled secondary antibodies (sc-2005, 1:10,000, Santa Cruz Biotechnology, Dallas, TX, USA). The signal was detected by chemiluminescence with Clarity Western ECL Substrate (Bio-Rad, Hercules, CA, USA) using ChemiDoc™ Imaging Systems (Bio-Rad, Hercules, CA, USA). Quantitative analysis was performed using ImageLabTM Software. The WB results were normalized using a stain-free technique, by measuring total protein directly on the WB membrane.

### 4.5. GFP Competition Assay

SeAx and HH cells were infected with miRZIP lentivirus, aiming at the infection percentage of 50%. The percentages of GFP-positive cells were measured using the flow cytometry (CytoFLEX S Flow Cytometer, Beckman Coulter, Indianapolis, IN, USA) on day 4 post-transduction and monitored tri-weekly for three weeks. Data were analyzed using Kaluza Analysis Software (Beckman Coulter, Indianapolis, IN USA). To determine the effect on cell growth, the percentage of GFP-positive cells on day 6 was set to 100%, and the fold difference relative to this starting point was calculated for each time point. To determine significant differences in the GFP assays, we used mixed model analysis as described previously [27].

### 4.6. Apoptosis Assay

The percentages of apoptotic cells were determined in SeAx and HH cells harvested on day 8 after transduction with the lentiviral miRZIP vectors aiming at an infection percentage of >95%. Briefly, cells were washed twice with cold phosphate-buffered saline and resuspended at a concentration of 1 × 10^6^ cells/mL in 1X Binding Buffer. Cells were stained with Annexin V APC and 7AAD according to the manufacturer’s protocol (BD Biosciences, San Jose, CA, USA) and analyzed via flow cytometry (CytoFLEX S Flow Cytometer, Beckman Coulter, Indianapolis, IN, USA).

### 4.7. RACE-PCR

Both 5′- and 3′-rapid amplification of cDNA ends (RACE) were performed using the SMARTer^®^ RACE 5′/3′ kit (Takara Bio Inc., San Jose, CA, USA), according to the manufacturer’s instructions. Briefly, 1 µg of total RNA isolated from the SeAx cell line was converted into the RACE-Ready first-strand cDNA. For the preparation of 5′-RACE-Ready cDNA/3′-RACE-Ready cDNA, the 5′-CDS Primer A/3′-CDS Primer A, respectively, were mixed with RNA. Two rounds of PCR amplification were performed, the first one with a specific primer 10XUPM (universal primer mix) and a gene-specific primer: TMEM244r primer for the 3′RACE and TMEM244f primer for the 5′RACE. To perform nested PCR, Universal Primer Short (UPM short) was added, as well as TMEM244r2 inner primer for the 3′RACE and TMEM244f2 inner primer for the 5′RACE. PCR products were analyzed using 1% agarose gel. Prior to qRT-PCR, the PCR products were purified using the QIAquick Gel Extraction Kit (Qiagen, Hilden, Germany). Subsequently, colony PCR was performed using RedTaq Polymerase (Merck KGaA, Darmstadt, Germany) and M13f, M13r primers. Different size bands were sequenced to identify the possible isoforms. Isoforms detected using RACE were confirmed using RT-PCR and primers designed to identify isoforms. The GAPDH gene was used as a positive control. All primer sequences are listed in Table S1.

### 4.8. RNA Extraction and Real-Time Quantitative PCR (RT-qPCR)

RNA was extracted using TRI Reagent (Merck KGaA, Darmstadt, Germany) according to the manufacturer’s protocol. The quantity of RNA was measured using the NanoDrop 2000 spectrophotometer (Thermo Fisher Scientific™, Waltham, MA, USA), and the quality was determined by 0.8% agarose gel electrophoresis with ethidium bromide staining. cDNA was synthesized from 0.3 µg or 0.5 μg of RNA using SuperScript™ IV Reverse Transcriptase with random hexamer primers (Invitrogen™, Waltham, MA, USA). *TMEM244* expression was analyzed using TaqMan Gene Expression Assays (Applied Biosystems, Foster City, CA, USA) (Hs02340633_m1) with intron-spanning primers located in the second and third exons. Beta-2 microglobulin (B2M) (Hs00984230_m1), with intron-spanning primers located in the first and second exons, was used as a reference gene for sample normalization. Relative gene expression was calculated using the median ct value method (2^−∆CT^). Expression levels of different isoforms were measured using 5× HOT FIREPol^®^ EvaGreen^®^ qPCR Supermix (Solis Biodyne, Tartu, Estonia) and primers specific to each isoform. The results were normalized to the *GAPDH* reference gene. Standard curves for each isoform were prepared as follows: PCR products for each *TMEM244* isoform and *GAPDH* gene were cloned into the pGEM^®^-T Easy Vector and transformed into bacteria. Vectors were isolated using the GeneJET Plasmid Miniprep Kit (Thermo Fisher Scientific™, Waltham, MA, USA), sequenced, and digested with the BstXI enzyme (NEB, Ipswich, MA, USA). Serial dilutions were prepared to obtain the concentration from 10^9^ to 10^1^ copy numbers. RT-qPCR was performed using the CFX96 Touch Real-Time PCR Detection System (Bio-Rad, Hercules, CA, USA).

### 4.9. Isolation of Nuclear and Cytoplasmic RNA

Cytoplasmic, nuclear, and chromatin RNA were isolated using an adaptation of the CD4+ T-cell nuclei extraction by Danko et al. [28,29]. RNA from the isolated fractions was reverse transcribed and used for qRT- PCR as described above. All samples were tested for *TMEM244* as well as tRNA lys, RPPH1, DANCER (cytoplasmic controls), U3SNORNA, ANRIL (nuclear controlss), and KTN1_AS1, KTN1_AS1_intron (chromatin controls). The sum of the cytoplasmic, nuclear, and chromatin expression levels of each transcript was set to 100%, and the percentage of each transcript localized to each compartment was determined. All controls showed the expected localization in each experiment, confirming successful fractionation. The primers used for RT-qPCR are listed in Table S1.

## 5. Conclusions

Our study is the first to experimentally verify the presence of TMEM244 protein. Different *TMEM244* transcript variants were identified; however, none of them had significant coding potential and they were all expressed at a lower level compared to the main transcript variant. Although *TMEM244* transcripts are localized in the cytoplasm, it appears that they do not encode a protein but are, rather, lncRNAs. Obtained results demonstrate that *TMEM244* mRNA is necessary for cellular growth of CTCL cells; therefore, it might be considered a new therapeutic target for the treatment of CTCL. Further study is needed to elucidate the in vivo effect and the downstream signaling pathway through which *TMEM244* functions in CTCL cells, as well as the function of its novel transcript variants.

## Data Availability

Data are available at request from the authors.

## Supplementary Materials

The following supporting information can be downloaded at: https://www.mdpi.com/article/10.3390/ijms24043531/s1.
