# Supplementary material for: TMEM244 Is a Long Non-Coding RNA Necessary for CTCL Cell Growth
Source: Int J Mol Sci. 2023 Feb 9;24(4):3531. doi: 10.3390/ijms24043531 (PMC9963807; doi:10.3390/ijms24043531)
Supplement: Supplementary file 1 [file ijms-24-03531-s001.zip › ijms-2086759-supplementary.pdf]

Supplementary Materials

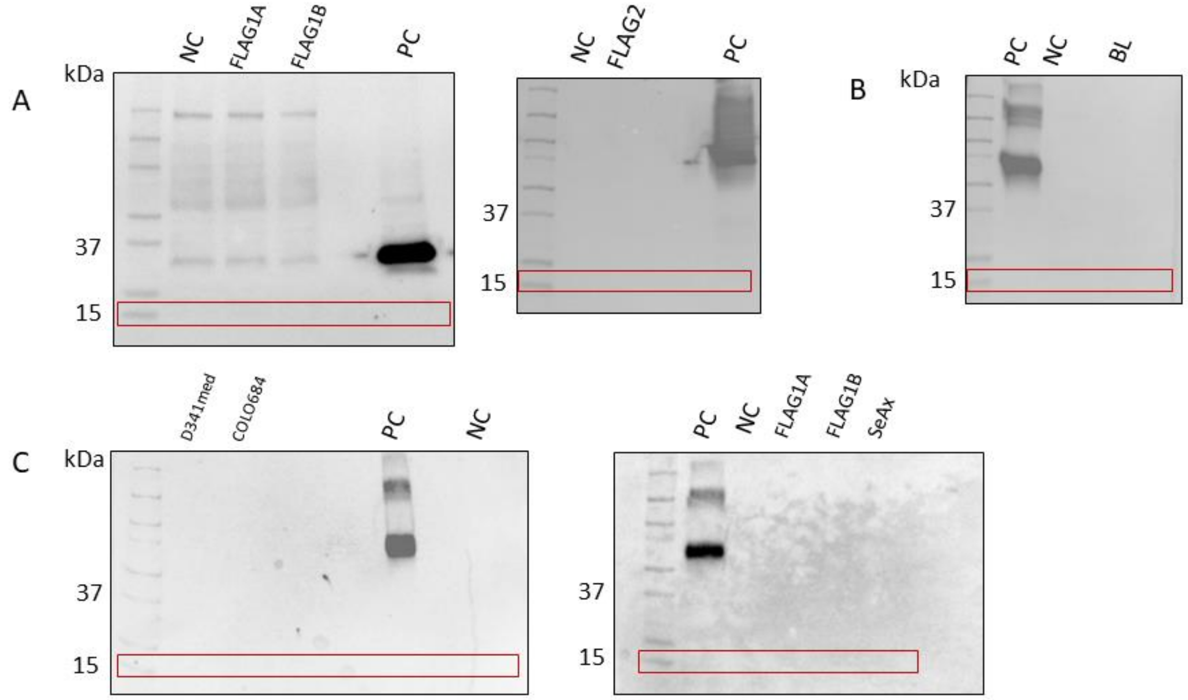

Figure S1. Western blot analysis of TMEM244 expression. Predicted protein size: TMEM244- 15kDa, TMEM244-flag- 16kDa. (a). Western blot analysis using anti-flag antibody. NC- negative control (Jurkat WT), PC- positive control (protein with flag- tag), FLAG1A- Jurkat pLV-CMV-Tmem244_flag, FLAG1B- Jurkat pLV-PGK_Tmem244_flag, FLAG2 – Jurkat pLV_flag_CMV_Tmem244. (b) Western blot analysis using anti-TMEM244 custom made antibody in brain lysate. PC- positive control (peptide used for anti-TMEM244 ab production), NC- negative control (Jurkat WT), B L- brain lysate. (c) Western blot analysis using anti-TMEM244 custom made antibody in cell lines. PC- positive control (peptide used for anti-TMEM244 ab production), FLAG1A- Jurkat pLV-CMV-Tmem244_flag, FLAG1B- Jurkat pLV-PGK_Tmem244_flag, NC- negative control (Jurkat WT).

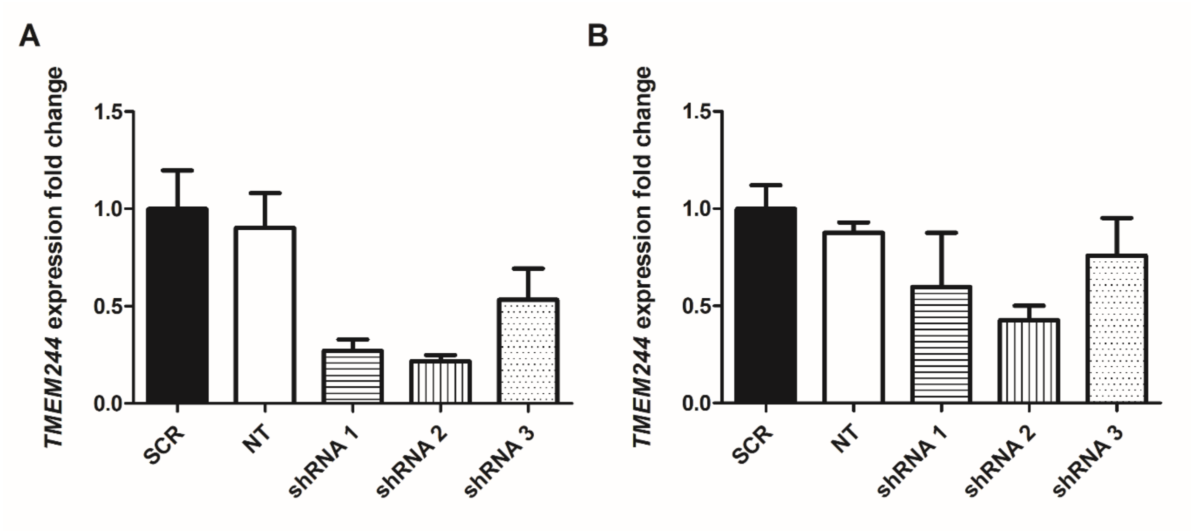

Figure S2. Confirmation of *TMEM244* expression knockdown by qRT-PCR in cell lines. (a) in SeAx cell line; (b) in HH cell line; SCR- scrambled, NT- non-targeting.

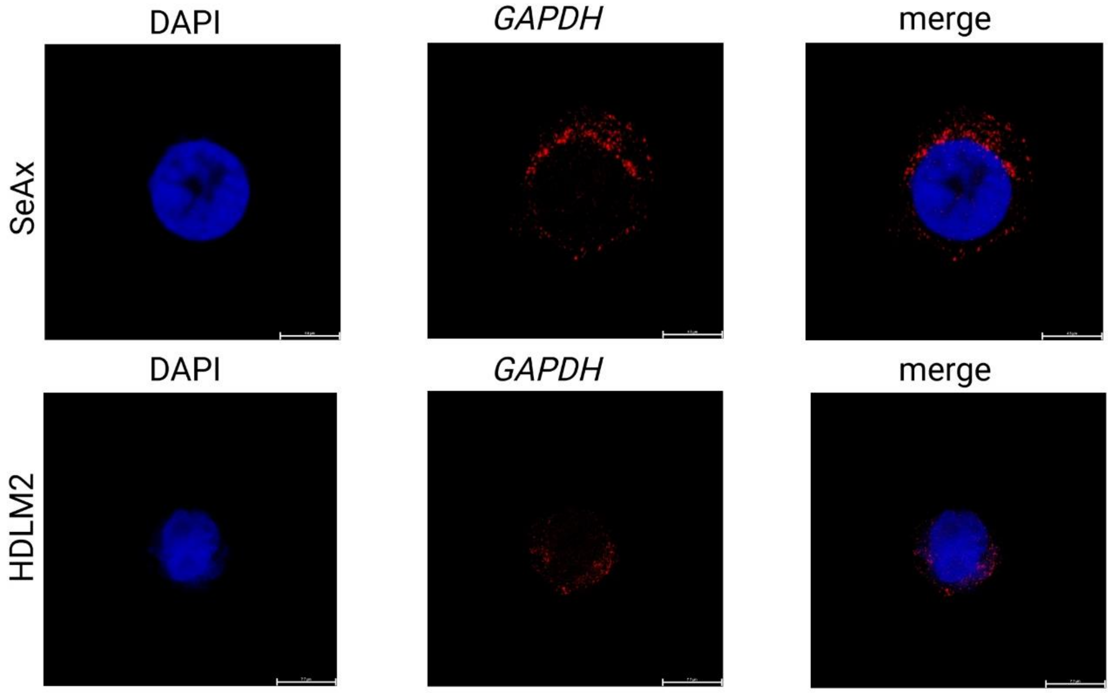

Figure S3. FISH analysis of GAPDH in SeAx and HDLM2 cells; GAPDH FISH signal in red, DAPI counterstain in blue.

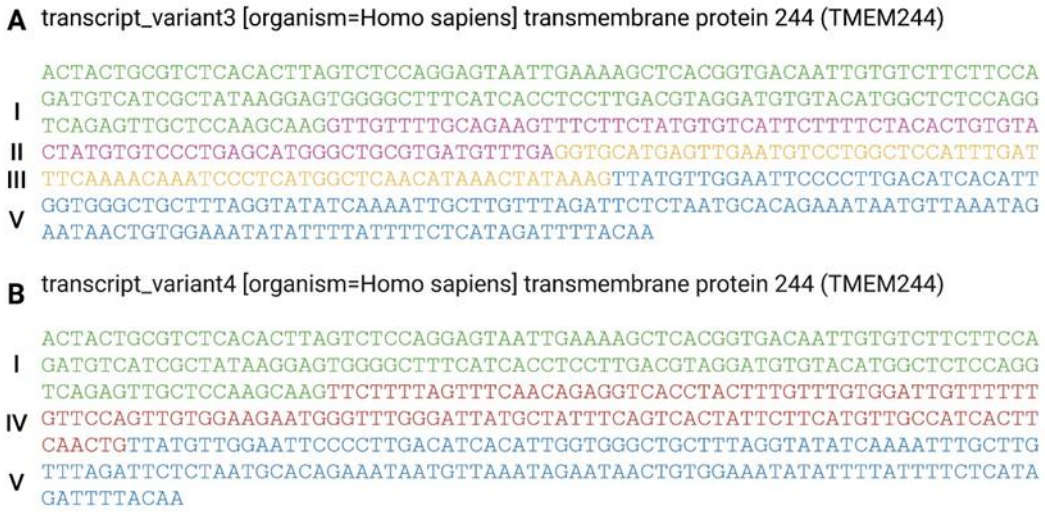

Figure S4. Sequences of identified *TMEM244* transcript variant: (a) transcript variant 3; (b) transcript variant 4. Green underline- exon 1; pink underline- exon 2; yellow underline- exon 3; red underline- exon 4; blue underline- exon 5.

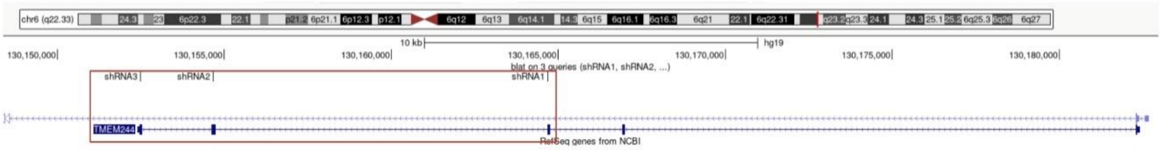

Figure S5. Schematic ilustration of the binding site for the shRNAs in the TMEM244 mRNA.

Table S1. The list of primers used in this study.

Primer name	Sequence (5′-3′)
Primers for RACE analysis	
TMEM244r	AGCAATTTTGATATACCTAAAGCAGCCCAC
TMEM244f	CACGGTGACAATTGTGTCTTCTTCCAG
TMEM244r2	CTAAAGCAGCCCACCAATGTGATGTC
TMEM244f2	GCTATAAGGAGTGGGGCTTTCATCAC
Ex1f	GACAATTGTGTCTTCTTCCAG
Ex5r	CTATGAGAAAATAAAATATATTTCCACAG
Ex3,5f	AAAGTTATGTTGGAATTCCCC
Ex1,4f	GTTGCTCCAAGCAAGTTCTTTTAG
Ex0f	CACGCTTCAACATGAATGCAG
Ex4f	CAGTTGTGGAAGAATGGGTTTG
GAPDHf	GACAGTCAGCCGCATCTTCT
GAPDHr	GCGCCCAATACGACCAAATC
M13 f	GTAAAACGACGGCCAG
M13 r	CAGGAAACAGCTATGAC
Primers for gene expression analysis by quantitative Real Time PCR	
tRNAlys_f	CGGCTAGCTCAGTCGGTAGA
tRNAlys_r	CCAACGTGGGGCTCGAAC
RPPHf	AGCTTGGAACAGACTCACGG
RPPHr	AATGGGCGGAGGAGAGTAGT
DANCERf	CGTCTCTTACGTCTGCGGAA
DANCERr	TGGCTTGTGCCTGTAGTTGT
U3SNORNAf	AACCCCGAGGAAGAGAGGTA
U3SNORNAr	CACTCCCCAATACGGAGAGA
ANRILf	AAGCCGCTCCGCTCCTCTTCT
ANRILr	GCCGTGTCCAGATGTCGCGT
KTN1_AS1f	GCAAAGACACAAGGCTCACA
KTN1_AS1r	ATGGTATTGGGGCACGTACA
KTN1_AS1_intronf	TTGGCTGCTATTTACTACCCTCC
KTN1_AS1_intronr	GCTGGGTGTGTTGCTAATCC
TMEM244f	GCTATAAGGAGTGGGGCTTTCATCAC
TMEM244r	CTAAAGCAGCCCACCAATGTGATGC

Table S2. The list of Stellaris® RNA FISH probes for *TMEM244* transcript used in this study.

Probe	Probe (5'-> 3')
1	GAAGACACAATTGTCACCGTG
2	CTTATAGCGATGACATCTGGA
3	CGTCAAGGAGGTGATGAAAGC
4	CTGGAGAGCCATGTACACATC
5	AACAACCTTGCTTGGAGCAAC
6	CACATAGAAGAAACTTCTGCA
7	GGACACATAGTACACAGTGTA
8	ATGCACCTCAAACATCACGCA
9	ATGGAGCCAGGACATTCAACT
10	GAGGGATTTGTTTTGAAATCA
11	CTTTATAGTTTATGTTGAGCC
12	GGTGACCTCTGTTGAAACTAA
13	TTCTTCCACAACTGGAACAAA
14	AAATAGCATAATCCCAAACCC
15	GGCAACATGAAGAATAGTGAC
16	CCAACATAACAGTTGAAGTGA
17	CCAATGTGATGTCAAGGGGAA
18	TTTGATATACCTAAAGCAGCC
19	TTTCTGTGCATTAGAGAATCT
20	ATTTCCACAGTTATTCTATTT
